# Supplementary material for: Low-Dose-Rate Irradiation Suppresses the Expression of Cell Cycle-Related Genes, Resulting in Modification of Sensitivity to Anti-Cancer Drugs
Source: Cells. 2022 Jan 31;11(3):501. doi: 10.3390/cells11030501 (PMC8833988; doi:10.3390/cells11030501)
Supplement: Supplementary file 1 [file cells-11-00501-s001.zip › cells-1536895-supplementary.pdf]

Supplementary Materials

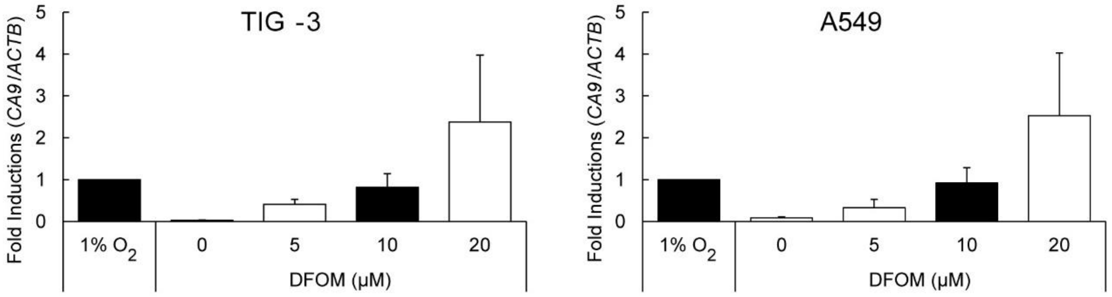

Figure S1. Optimization of DFOM treatment in TIG-3 and A549 cells. The appropriate amount of DFOM required to mimic hypoxia for each cell type was determined by evaluating the ability of DFOM upon HIF-1 activation to induce a target gene, *CA9*, in each cell, compared with hypoxia (1% O_2). TIG-3 and A549 were subjected to hypoxia or various concentrations of DFOM for 24 hours. The bar graphs show fold inductions of *CA9* expression. The condition 1% O_2 is considered the control; black bars indicate similar ability of HIF-1 activation under DFOM treatment. Bars are means and whiskers are SD (n =3).

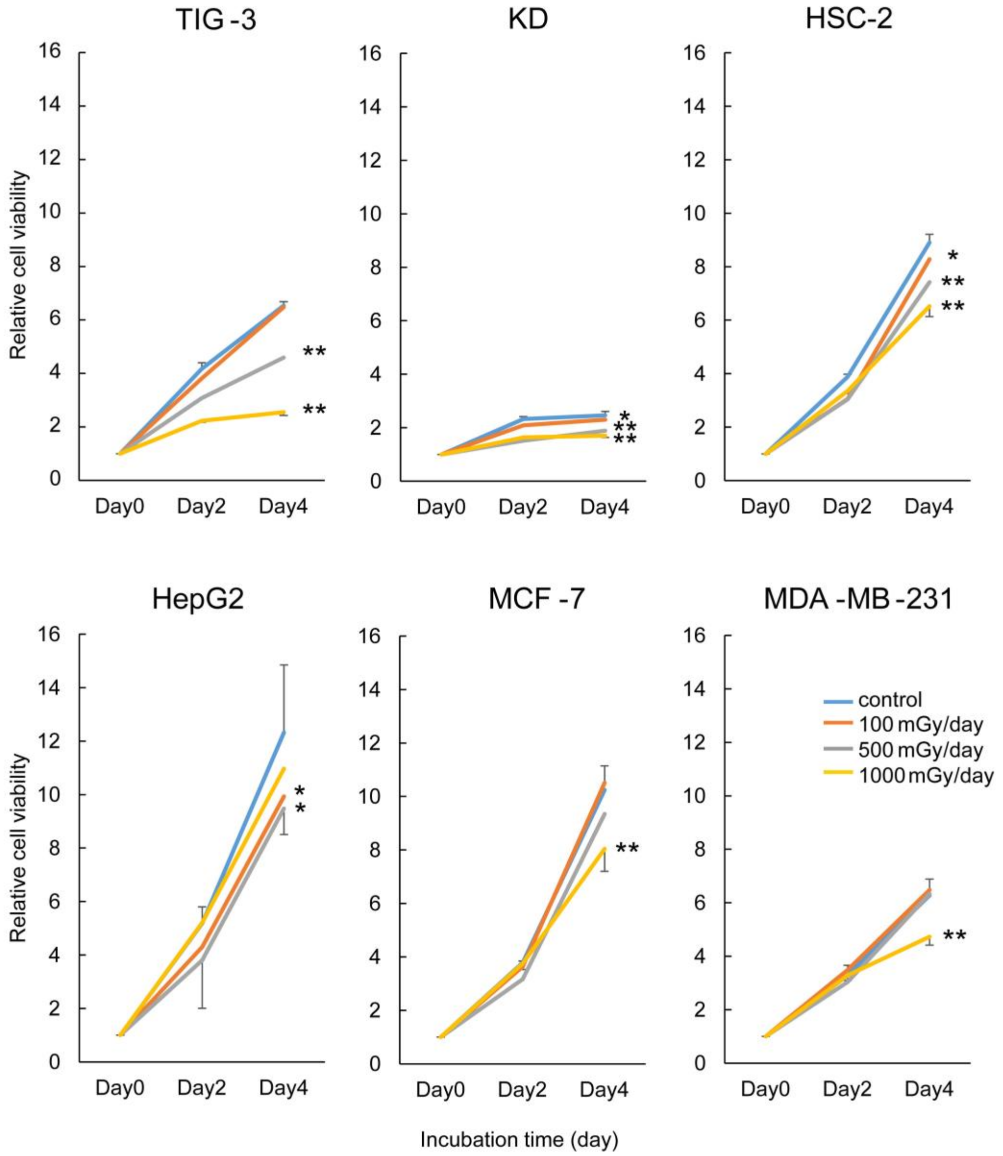

Figure S2. Effects of LDR-IR on cell proliferation. The relative cell viabilities of human lung fibroblast (TIG-3), oral fibroblast (KD), oral squamous carcinoma (HSC-2), hepatoblastoma (HepG2), and breast cancer (MCF-7 and MDA-MB-231) under normoxic conditions were evaluated by couting cell numbers after LDR-IR (day 0, 2, 4). Values are mean and SD (n = 3); *P < 0.05; **P < 0.01 vs. control.

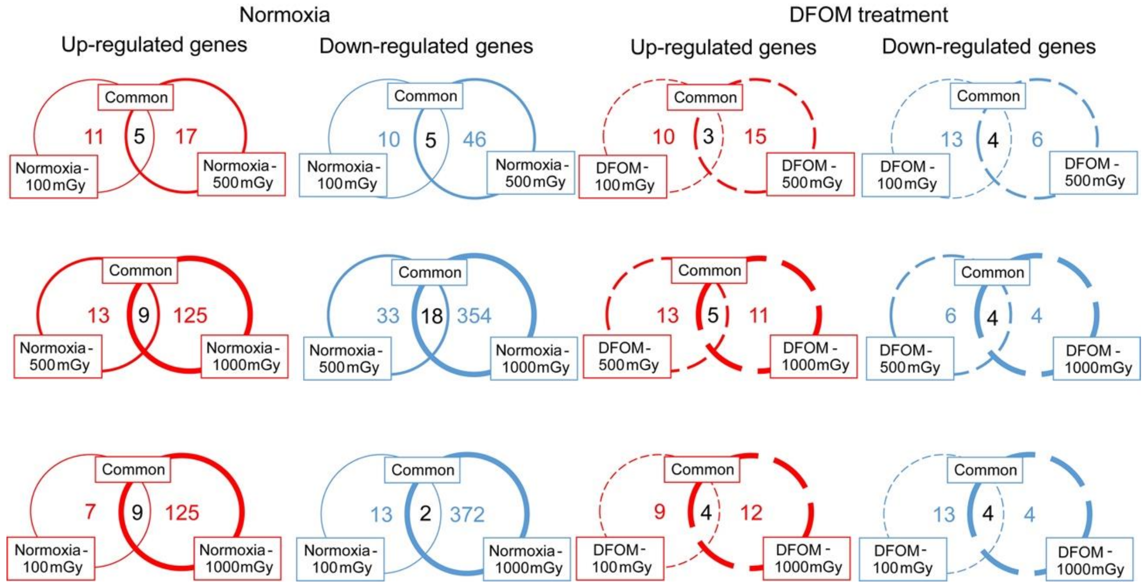

Figure S3. Venn diagram illustrating differentially expressed genes in LDR-irradiated cells. Up-regulated (red) or down-regulated (blue) genes are shown for each dose of LDR irradiation under normoxia or DFOM treatment are shown as indicated.

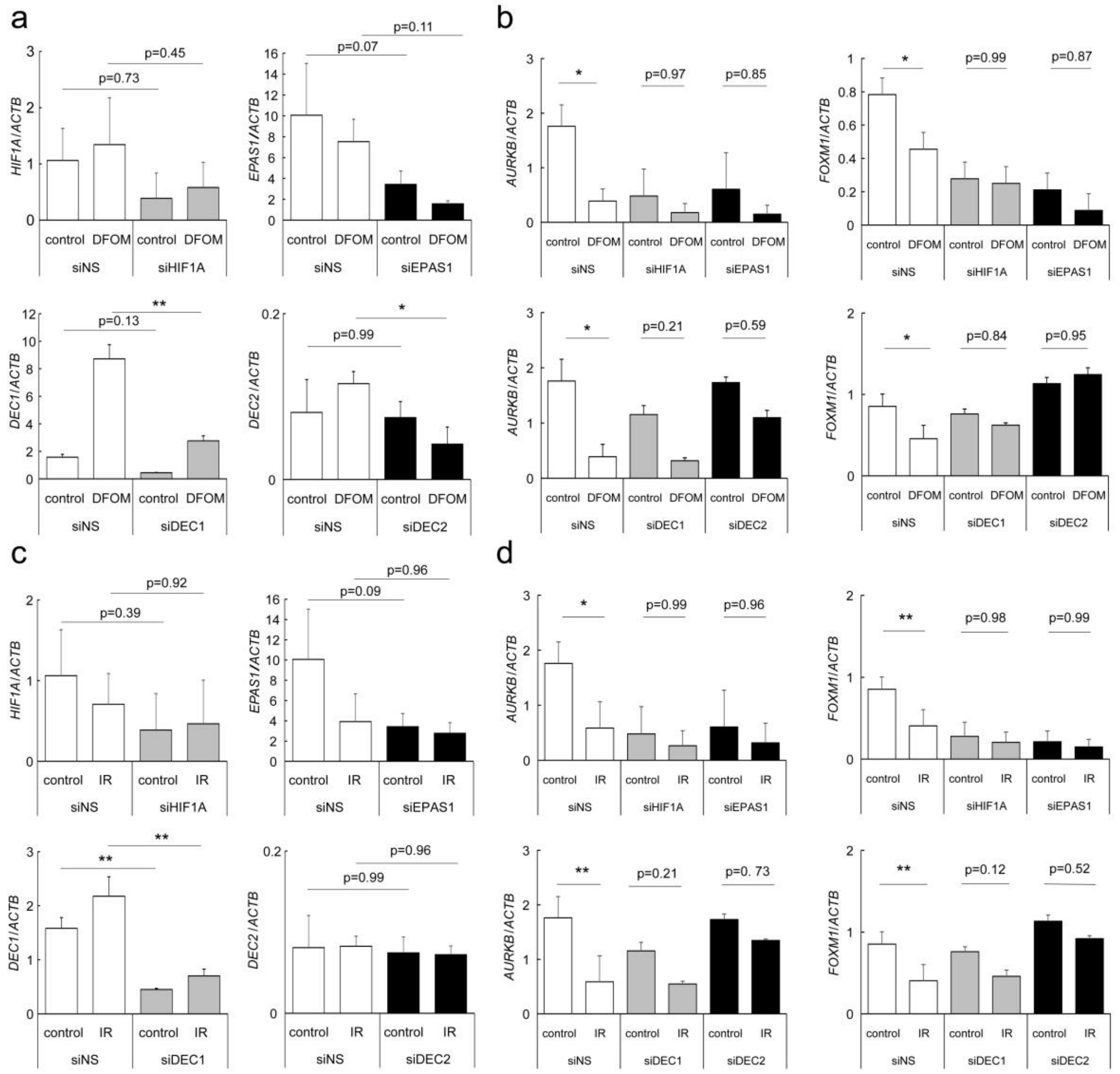

Figure S4. Mechanisms of regulation of *AURKB* and *FOXM1* gene expression in A549 cells. Knockdown experiments were performed by using transient transfectons with siRNA specific for *HIF1A*, *EPAS1*, *DEC1*, and *DEC2* genes. Expression level of *HIF1A*, *EPAS1*, *DEC1*, and *DEC2* in A549 cells treated with DFOM (a) or radiation (IR: 1000 mGy/day) (c). Expression level of *AURKB* and *FOXM1* in A549 cells treated with DFOM (b) or radiation (IR: 1000 mGy/day) (d). Expression levels were analyzed with quantitative RT-PCR. Relative gene expression levels were calculated as the ratio to that of *ACTB*, and are represented as mean (bars) and SD (whiskers; n =3). *$P < 0.05$; **$P < 0.01$.

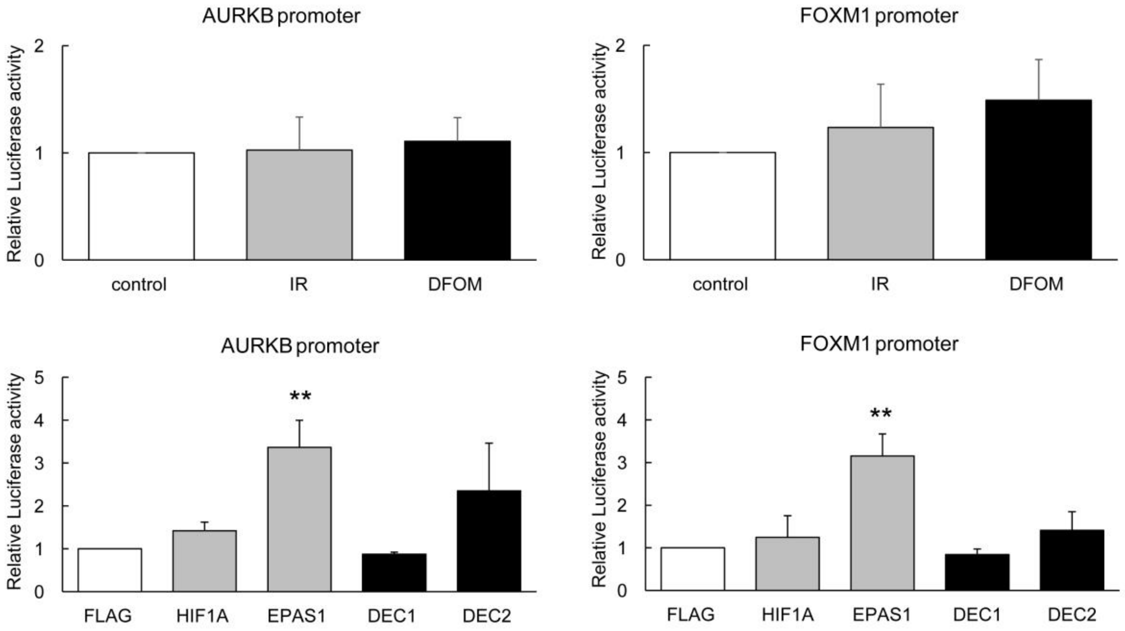

Figure S5. Effects of LDR-IR, DFOM treatment, or co-transfection with hypoxia-regulated transcription factor genes on *AURKB* and *FOXM1* promoter activity. The 5′ promoter region of *AURKB* and *FOXM1* was subcloned into pGL4.26 luciferase reporter plasmid to generate the AURKB and FOXM1 promoter reporters (pGL4.26-AURKB and pGL4.26-FOXM1). Upper panel shows the relative luciferase activity in TIG-3 cells under normoxia (control), IR (1000 mGy/day), or DFOM treatment. Lower panel shows the relative luciferase activity in TIG-3 cells co-transfected with hypoxia-regulated transcription factor gene (*HIF1A*, *EPAS1*, *DEC1*, and *DEC2*) expression vectors. Relative firefly luciferase activity was calculated as the ratio to that of renilla luciferase activity as a transfection efficiency control, and are represented as mean (bars) and SD (whiskers; n =3). **P < 0.01 vs. FLAG.

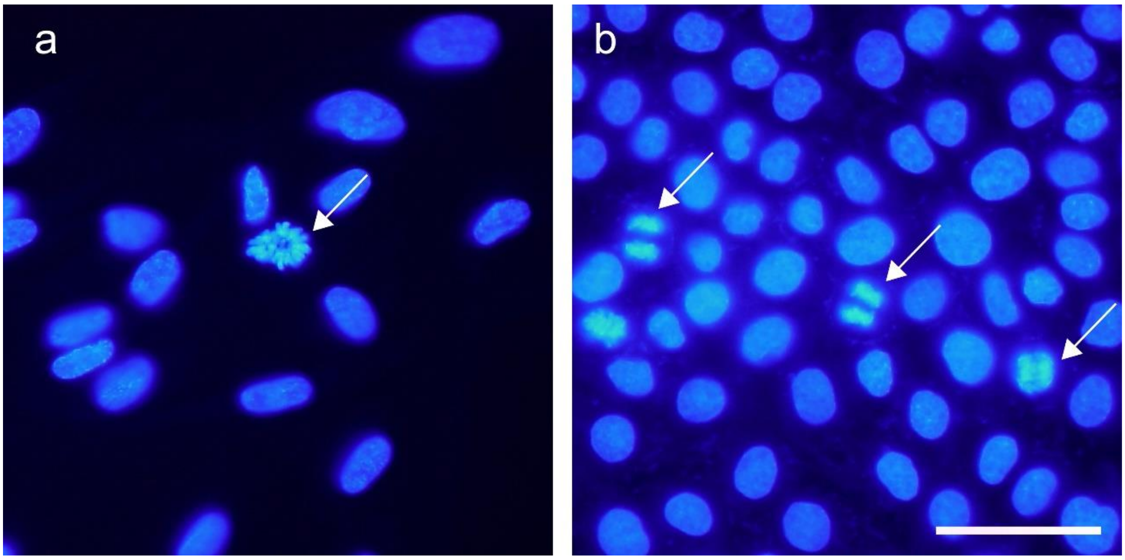

Figure S6. Effects of LDR-IR on nuclear morphology. Representative images of TIG-3 (a) and A549 (b) cells are shown. Fluorescence shows DAPI nuclear staining (blue) and cells in M phase are indicated with white arrow. Scale bar = 50 μm (a, b).

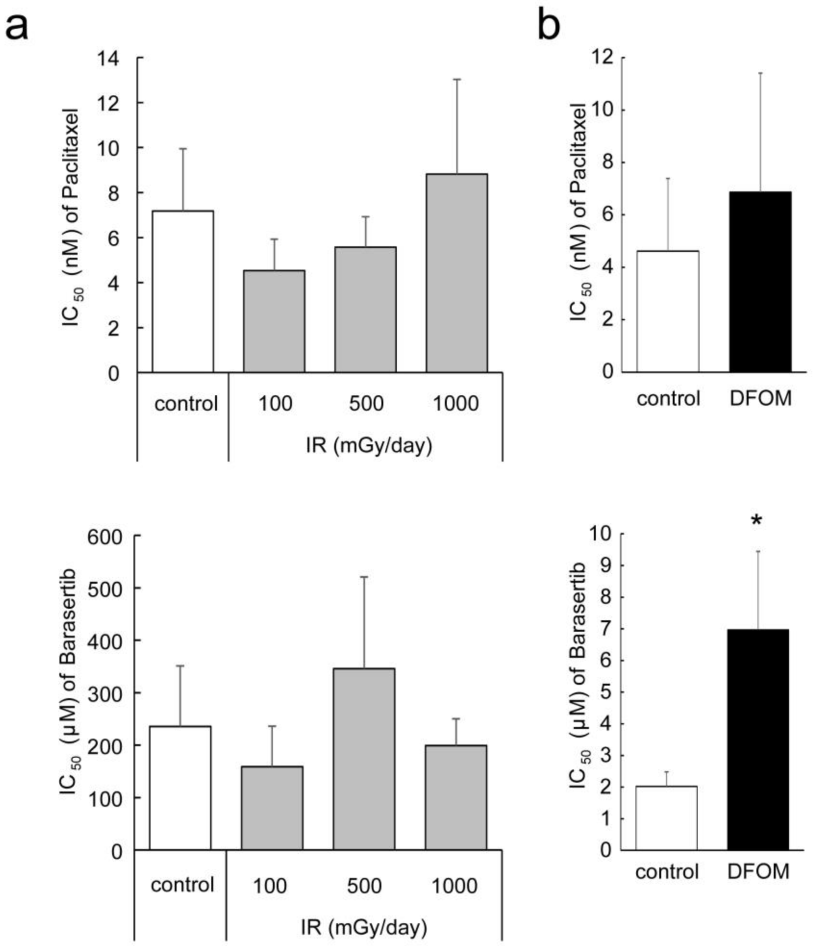

Figure S7. Effect of LDR-IR on sensitivity to cell-cycle-targeting anti-cancer drugs in HSC-2 cells. IC_{50} values in response to Paclitaxel and Barasertib in HSC-2 cells pre-treated with LDR-IR (n = 4) (a) or DFOM (n = 5) (b) were evaluated with the MTT assay. (a, b): Values are mean and SD. *$P < 0.05$ vs. control.

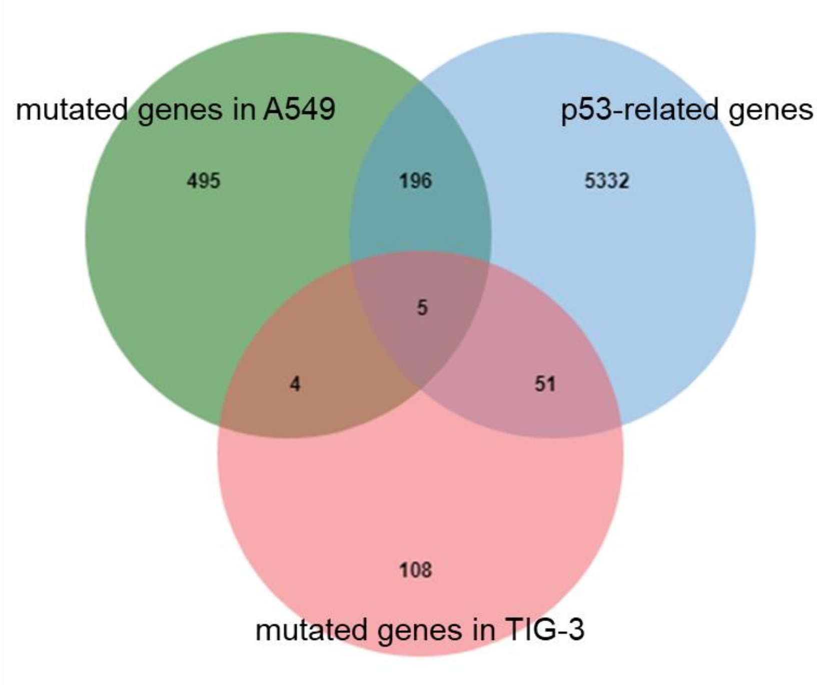

Figure S8. Venn diagram illustrating mutation spectrum of the p53-related gene in A549 and TIG-3 cells. A set of p53-related genes was cited from Harmonizome (https://maayanlab.cloud/Harmonizome/), and mutated genes in A549 and TIG-3 cells were identified by COSMIC (https://cancer.sanger.ac.uk/cosmic). Numbers of overlapped genes are indicated.

Table S1. Primer and probe sets for quantitative RT-PCR

ARUKB-F: 5'- CTCCTACCCCTGGCCCTAC-3'
ARUKB-R: 5'- AGGCTCTTTCCGGAGGACT-3'
ARUKB-probe: UPL #43 (Roche)

FOXM1-F: 5'- GCAGCATCAAGCAAGAGATG-3'
FOXM1-R: 5'- GACGCTGATGGTCTCGAAG-3'
FOXM1-probe: UPL #1 (Roche)

HIF1A-F: 5'- GAACCTGATGCTTTAACTTTGCT-3'
HIF1A-R: 5'- TGCTGGTCATCAGTTTCTGTG-3'
HIF1A-probe: UPL #28 (Roche)

EPAS1-F: 5'- GACATGAAGTTCACCTACTGTGATG-3'
EPAS1-R: 5'- GCGCATGGTAGAATTCATAGG-3'
EPAS1-probe: UPL #17 (Roche)

CA9-F: 5'- CCTTTGCCAGAGTTGACGAG-3'
CA9-R: 5'- GCAACTGCTCATAGGCACTG-3'
CA9-probe: UPL #25 (Roche)

DEC1-F: 5'- GACTGGAGCACGGAGACCT-3'
DEC1-R: 5'- GGTGCGGCAATTTGTAGG-3'
DEC1-probe: UPL #56 (Roche)

DEC2-F: 5'- CTACTGCGTGCCCGTCAT-3'
DEC2-R: 5'- CGGTGTCCGTGTCGTTCT-3'
DEC2-probe: UPL #26 (Roche)

BAX-F: 5'- CCATCATGGGCTGGACAT-3'
BAX-R: 5'- CACTCCCGCCACAAAGAT-3'
BAX-probe: UPL #69 (Roche)

BCL2-F: 5'- TTGGTATCCTTCTCTTTCACGCAC-3'
BCL2-R: 5'-ATGGCATTGACGAAGAGGAT -3'
BCL2-probe: UPL #23 (Roche)
